# Improved antioxidant status by omega-3 fatty acid supplementation in breast cancer patients undergoing chemotherapy: a case series

**DOI:** 10.1186/s13256-015-0619-3

**Published:** 2015-06-24

**Authors:** Prakash Mansara, Mrunal Ketkar, Rashmi Deshpande, Amol Chaudhary, Kavita Shinde, Ruchika Kaul-Ghanekar

**Affiliations:** 1 Interactive Research School for Health Affairs (IRSHA), Bharati Vidyapeeth Deemed University, Katraj-Dhankawadi, Pune, 411043 Maharashtra India; 2 Department of Surgery, Bharati Vidyapeeth Hospital and Research Centre (BVHRC), Pune, 411043 India

**Keywords:** Catalase, Chemotherapy, Fish oil, Glutathione reductase, Plasma antioxidant, Superoxide dismutase

## Abstract

**Introduction:**

Breast cancer is the second leading cause of cancer death in women worldwide and the third most common cancer in India. Various studies have reported that chemotherapy reduces the antioxidant status in patients with cancer. A diet rich in omega-3 fatty acids has been shown to offer protection against breast cancer through various mechanisms. However, there are no reports suggesting a relationship between consumption of omega-3 fatty acids during chemotherapy and antioxidant status in patients with breast cancer. Thus, the objective of this study was to evaluate whether fish oil supplementation could improve the antioxidant status of five women with breast cancer undergoing chemotherapy.

**Case presentation:**

We report on the cases of five Indian women with breast cancer, in the age group of 34 to 60 years, who had poorly differentiated breast carcinoma and underwent modified radical mastectomy. Postsurgery, the patients were given fish oil capsules containing eicosapentaenoic acid (180mg) and docosahexaenoic acid (120mg)/capsule during their chemotherapy. Informed consent was obtained from each participant and they were followed-up to the completion of six chemotherapy cycles at 21-day intervals.

**Conclusions:**

The supplementation of fish oil significantly (*p* < 0.01) increased superoxide dismutases, glutathione reductase and catalase activity in red blood cells as well as the total plasma antioxidant status in the patients. This approach of using omega-3 fatty acids as an adjuvant treatment for breast cancer may help oncologists to manage the side effects of ongoing chemotherapy by improving the antioxidant status in patients.

## Introduction

Breast cancer is the third most common cancer in Indian women, followed by cervical and stomach cancers [1]. The role of nutrition in the prevention of cancer has been recently established based on the available epidemiological data. Dietary agents have been shown to suppress the transformative, hyperproliferative and inflammatory processes that initiate carcinogenesis [2].

Dietary fatty acids have been shown to play an important role not only in normal growth and development but also in the treatment of cancer and other diseases [3, 4]. Various studies have reported the anti-cancer activity of omega-3 polyunsaturated fatty acids (n-3 PUFAs), eicosapentaenoic acid (EPA) and docosahexaenoic acid (DHA), usually found in cold water fish. Clinical studies have shown that these fatty acids benefitted patients with cancer by; increasing their response to chemotherapy; lowering the side effects of chemotherapy; increasing survival; improving quality of life (QoL); reducing hospital stay and severity of postoperative infections; and improving appetite, body weight, nutrition and clinical performance parameters such as liver and pancreas function [4–7]. EPA and DHA have been reported to improve the therapeutic index of chemotherapeutic drugs such as anthracyclines, purine and pyrimidine analogues, alkylating agents, cisplatin and vinca alkaloids by either increasing their efficacy and/or reducing their toxicity in different cancers, including breast cancer [5, 8]. However, to date the effect of the consumption of fish oil (FO) by patients with breast cancer undergoing chemotherapy on their antioxidant status has not been explored.

## Case presentation

The present study was carried out at our hospital, 2 years ago for a period of 1 year and 9 months and was approved by the Institutional Ethical Committee. Informed consent was obtained from all the participants in the study. The patients were negative for human immunodeficiency virus, hepatitis, pancreatitis, iron deficiency anemia or renal disease. All the patients underwent standard preoperative malignancy investigations including mammography, ultrasound examination and fine needle aspiration cytology (FNAC) of the lesion. The results showed poorly differentiated breast carcinoma in the patients who were subsequently advised for modified radical mastectomy (MRM). Preoperative X-ray chest posterior-anterior (PA) view and ultrasonography (USG) scan of the abdomen showed no distant metastasis at the time of admission. No episodes of bleeding, failure of sutures, infection or death were reported after surgery. The patients were followed-up during the entire course of six chemotherapy cycles. Patients were given FO during chemotherapy in the form of Mega-3™ capsules (Dr. Reddy’s Laboratories), containing EPA (180mg) and DHA (120mg)/capsule. Since we wanted to evaluate the antioxidant potential of only FO, we selected Mega-3™ capsules as they did not contain any other component, particularly antioxidants. The dose for each patient was decided by the physician based on the grade of the cancer and the patient’s overall clinical condition. For Cases 2 to 5, six capsules per day were given and for Case 1, four capsules per day were given. The dosage in all the cases did not exceed more than 2 g per day and was according to US Food and Drug Administration recommendations [9].

Brief details of each case are presented below and the patients’ characteristics are summarized in Table 1.

**Table 1:** Clinical characteristics of the patients with breast cancer

Patient	Case 1	Case 2	Case 3	Case 4	Case 5
Age (years)	34	60	51	59	45
BMI	23.7	19.6	25.1	17.8	30.7
Age at first period (years)	14	15	15	13	14
Age at first birth (years)	18	17	17	18	18
ECOG score [21]	1	3	2	2	3
Preoperative lump size (cm)	4 × 3	5 × 3	6 × 4	4 × 2	10 × 8
TNM Stage	T_1_N_0_M_X_	T_4_N_0_M_0_	T_2_N_1_M_0_	T_2_N_0_M_0_	T_2_N_2_M_X_
ER	Negative	Positive (80 %)	Positive (90 %)	Positive (80 %)	Negative
PR	Negative	Positive (80 %)	Positive (85 %)	Negative	Negative
HER2	Positive (score: 3+)	Negative	Negative	Negative	Positive (score: 3+)
Fish oil dosage (gm/day)	1.2	1.8	1.8	1.8	1.8
No. of days of fish oil supplementation	130	130	188^a^	136	132
No. of capsules per day	Four	Six	Six	Six	Six
Chemotherapy regimen	Adriamycin (72mg/100ml/10min), 5-fluorouracil (725mg/100ml/10min), cyclophosphamide (725mg/500ml/10min)	Doxorubicin (60mg/100ml/10min), 5-fluorouracil (685mg/100ml/10min), cyclophosphamide (685mg/500ml/60min)	Doxorubicin (70mg/100ml/10min), 5-fluorouracil (700mg/100ml/10min), cyclophosphamide (700mg/500ml/60min)	Paclitaxel (220mg/500ml/180min)	Doxorubicin (90mg/100ml/10min), 5-fluorouracil (900mg/100ml/10min), cyclophosphamide (900mg/500ml/60min)

*BMI* body mass index, *ECOG* Eastern Cooperative Oncology Group, *ER* estrogen receptor, *PR* progesterone receptor, *HER2* human epidermal growth factor receptor

^a^The increase in the number of days was due to patient’s delay in undertaking chemotherapy on time because of her financial restrictions

### Case 1

Case 1 is an Indian woman who has been a tobacco user since the age of 12; her family history did not show any type of malignancy in her first degree relatives. She complained of a lump in her left breast which was painless, mobile and persistent in size. No significant surgical history was reported and she had normal weight as per her body mass index (BMI). On clinical examination, a hard lump was palpated in the upper medial quadrant of her left breast. Axillary lymph nodes were free of tumor.

### Case 2

Case 2 is an Indian woman was admitted with the chief complaint of a left breast lump persisting for 2 months, which had caused her pain for the last 15 to 20 days. She has been a tobacco user since the age of 15. She had a past medical history indicating bronchial asthma and had undergone lumpectomy approximately 8 years ago. She did not have any family history of cancer and had normal weight as per her BMI. She had osteopenia and high serum alkaline phosphatase (ALP) levels at the time of admission to our hospital. She was diagnosed with infiltrating ductal carcinoma.

### Case 3

Case 3 is an Indian woman who was admitted with the chief complaint of a right breast lump persisting for the last 2 months. It had caused her pain for the month before admission to our hospital and showed tenderness upon touch. No nipple discharge was observed. She has been a tobacco user since the age of 35. She had a past medical history of hysterectomy, approximately 30 years ago and had hypertension. She was a known case of rheumatic heart disease. She did not have any personal or family history of cancer and was overweight as per her BMI. She was diagnosed with infiltrating ductal carcinoma. She was reported to have decreased appetite upon admission.

### Case 4

Case 4 is an Indian woman who was admitted with the chief complaint of a right breast lump persisting for 6 months which was painless and had gradually increased in size. She had a past medical history of oophorectomy and had hypertension for 1 year. She did not have any family history of cancer and was underweight as per her BMI. She was reported to have decreased appetite and low hemoglobin at the time of admission. She was diagnosed with infiltrating ductal carcinoma.

### Case 5

Case 5 is an Indian woman who was admitted with the chief complaint of a left breast lump persisting for 2 to 3 months that was associated with nipple inversion. She had been a tobacco user since the age of 20. She did not have any family history of cancer. She belonged to obese class I as per her BMI and had been diagnosed with infiltrating ductal carcinoma.

We collected venous blood from all the five patients after surgery (but before chemotherapy and FO supplementation) and after every cycle of chemotherapy (during FO supplementation) in ethylenediaminetetraacetic acid (EDTA) coated vials. The blood was immediately layered onto the histopaque column (Sigma-Aldrich) and centrifuged at 400 × g for 35min to separate the red blood cells (RBCs) and plasma fractions. RBCs were washed with saline and together with plasma were stored at −80 °C until further analysis. The blood samples were analyzed for the status of antioxidant enzymes (SOD, CAT and GRx) in RBCs and total antioxidant status in plasma. SOD (EC 1.15.1.1), CAT (EC 1.11.1.6) and GRx (EC 1.6.4.2) were estimated by using commercially available kits (Cayman, Michigan, USA). Total plasma antioxidants were measured by oxygen radical absorbance capacity (ORAC) assay as described previously [10]. Patients filled out a self-administered European Organisation for Research and Treatment of Cancer (EORTC) QLQ-C30 [11] questionnaire before chemotherapy as well as after chemotherapy and FO supplementation. It is a multidimensional validated cancer-specific measure that includes global health status and QoL, functional and symptom scales. EORTC QLQ-C30 subscales were calculated according to the EORTC-QLQ manual and vary from 0 to 100. A high score for a functional or QoL scale represents high level of functioning or QoL. A high score for a symptom scale represents high level of problems. Statistical analysis was performed by using GraphPad Prism 5. The results were plotted after averaging out the data from all the chemotherapy cycles (after FO supplementation) for each patient and then plotted as a cumulative mean of the data obtained from all the five patients. All the results have been presented as mean ± standard deviation. The data was analyzed by using a two-tailed paired *t* test to compare the effect of FO intervention on antioxidant enzymes and total antioxidant status before and after supplementation, with a significance level of 0.05.

## Results

**Fig. 1:**
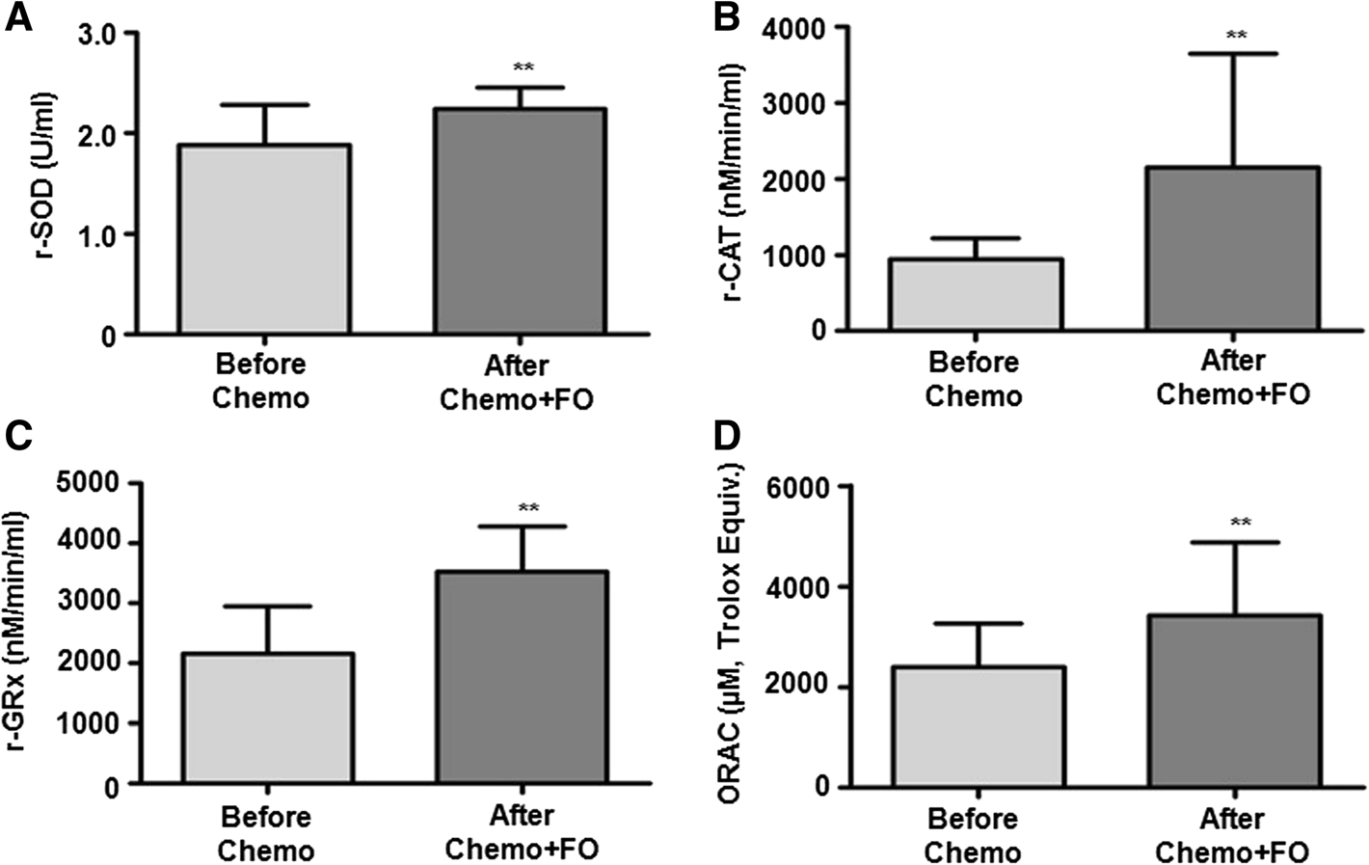
Fish oil supplementation improves the antioxidant status in five patients with breast cancer. Superoxide dismutase (*SOD*) (**a**), catalase (*CAT*) (**b**) and glutathione reductase (*GRx*) (**c**) activities in red blood cells and plasma antioxidant status (**d**) in the patients before and after chemotherapy and fish oil supplementation (*Chemo + FO*) have been shown. The results have been presented as mean ± standard deviation. **p < 0.01 indicates statistically significant difference in the results before and after fish oil intervention. *ORAC* oxygen radical absorbance capacity

The clinical characteristics of each patient including age, BMI and other parameters have been summarized in Table 1. The patients showed a significant increase (*p* < 0.01) in SOD, CAT and GRx levels in the RBC fractions after FO supplementation as compared to the values obtained before chemotherapy and FO supplementation (Fig. 1a–c).

We also evaluated the total antioxidant status in the plasma samples of all the patients by ORAC. The assay is based on the inhibition of free radical production from the 2,2′-azobis (2-amidinopropane) dihydrochloride (AAPH) substrate by the antioxidant molecules present in the test sample. It can determine both hydrophilic and lipophilic antioxidants, which represent the total antioxidant capacity. All the patients showed a significant increase in ORAC values (*p* < 0.01) after omega-3 fatty acid supplementation (Fig. 1d).

## Discussion

Various studies have reported a decrease in antioxidant levels and an increase in reactive oxygen species (ROS) in patients with breast cancer post-chemotherapy [12, 13]. Such reduced antioxidant status and enhanced ROS often lead to various side effects such as nephrotoxicity, cardiotoxicity and peripheral neuropathy that hamper tumor treatment and may even lead to a patient’s death. The antioxidant enzymes such as SOD, GPx and CAT work together in human cells to remove the toxic ROS. However, the role of omega-3 fatty acid supplementation in the modulation of antioxidant status has not been properly evaluated at the clinical level. In our study, all the patients were found to show significant improvement in their antioxidant status in RBC and plasma fractions after FO supplementation.

Recently, nutrition has become one of the most important aspects in cancer management. Reduced nutrition and subsequent poor health lowers antioxidant status, which may be associated with increased neoplastic activity in patients with cancer [14, 15]. An antioxidant rich diet has been shown to enhance the chemotherapy response and improve the QoL in patients by minimizing the side effects of chemotherapeutic drugs [14, 15]. Supplementation of vitamins C and/or E before and after breast cancer diagnosis, as well as radiation and hormone therapy, has been shown to protect against chemotherapy-related side effects through an increase in SOD, CAT, glutathione (GSH) and GRx and a decrease in the levels of malondialdehyde and DNA damage [15]. Similarly, the Shanghai Breast Cancer Survival Study has indicated that supplementation of vitamins E, C or multivitamins within 6 months of breast cancer diagnosis correlated with a decrease in recurrence rate and mortality [16]. Such supplementation may also alleviate dose-limiting toxicities which would help patients to complete the prescribed chemotherapy regimens resulting in better management of cancer.

The rationale behind supplementation of EPA and DHA in patients with breast cancer was laid down by various cross-sectional studies, which showed that higher intake of these fatty acids was associated with decreased risk of cancer-related mortality [17]. Other studies have shown that omega-3 fatty acids from fish/shell fish could decrease the risk of breast cancer and adverse events related to it [18]. FO supplementation has been shown to: reduce malignant epithelial cell proliferation (Ki67) marker in prostate cancer; reverse cachexia in advanced pancreatic cancer; maintain patient weight and muscle mass during chemotherapy in lung cancer; improve liver and pancreas function in postoperative patients with abdominal cancer; and increase chemotherapeutic efficacy (without affecting the toxicity profile) and survival in patients with lung cancer [5–8, 19]. Based on the EORTC questionnaire, after chemotherapy and FO supplementation, patients showed improved QoL scores compared to that observed before chemotherapy (Table 2). The patients showed significant improvement in global health status after supplementation of FO during chemotherapy. Global health status is an indication of a patient’s own judgment of health status and QoL [20]. The functional status scales that included physical, emotional and social functioning were found to be significantly improved after chemotherapy and FO supplementation. The patients showed significant reduction in fatigue, pain and appetite loss scale after receiving FO during chemotherapy. Other functional or symptom scales of EORTC QLQ-C30 did not differ significantly before chemotherapy and after chemotherapy and FO supplementation.

**Table 2:** Fish oil supplementation improved quality of life in five patients with breast cancer

	Before chemotherapy	After chemotherapy and fish oil supplementation	*P* value
EORTC QLQ-C30 Global health score	46.67 ± 5.00	86.67 ± 3.33	<0.001
EORTC QLQ-C30 Functional scales			
Physical functioning score	48.00 ± 3.89	77.33 ± 2.67	<0.001
Role functioning score	46.67 ± 8.17	80.00 ± 8.16	ns
Emotional functioning score	10.00 ± 3.12	53.33 ± 6.77	<0.001
Cognitive functioning score	80.00 ± 6.24	80.00 ± 8.16	ns
Social functioning score	33.33 ± 5.27	66.67 ± 10.54	<0.001
EORTC QLQ-C30 Symptom scales			
Fatigue score	62.22 ± 10.31	26.66 ± 6.67	<0.001
Nausea and vomiting score	30.00 ± 8.16	10.00 ± 4.08	ns
Pain score	56.67 ± 8.50	23.33 ± 8.50	<0.001
Dyspnoea score	53.33 ± 8.17	26.67 ± 12.47	ns
Insomnia score	53.33 ± 8.17	33.33 ± 0.00	ns
Appetite loss score	93.33 ± 6.67	26.66 ± 6.67	<0.001
Financial difficulties score	80.00 ± 13.33	73.33 ± 12.47	ns

*EORTC* European Organisation for Research and Treatment of Cancer, *ns* nonsignificant

## Conclusions

The supplementation of FO containing omega-3 fatty acids, EPA and DHA, benefitted patients with breast cancer by increasing their antioxidant levels. These results strongly suggest that FO should be tested in an adequate randomized study to elucidate its possible role as an adjuvant in the management of breast cancer.

## Consent

Written informed consent was obtained from the patients for publication of this case series. Copies of the written consents are available for review by the Editor-in-Chief of this journal.

## Abbreviations

BMI: Body mass index
CAT: Catalase
DHA: Docosahexaenoic acid
EORTC: European Organisation for Research and Treatment of Cancer
EPA: Eicosapentaenoic acid
FO: Fish oil
GRx: Glutathione reductase
ORAC: Oxygen radical absorbance capacity
QoL: Quality of life
RBC: Red blood cell
ROS: Reactive oxygen species
SOD: Superoxide dismutase
